# Working conditions, self-perceived stress, anxiety, depression and quality of life: A structural equation modelling approach

**DOI:** 10.1186/1471-2458-8-48

**Published:** 2008-02-06

**Authors:** Bin Nordin Rusli, Bin Abdin Edimansyah, Lin Naing

**Affiliations:** 1 Division of Occupational Medicine, Department of Community Medicine, School of Medical Sciences, Health Campus, Universiti Sains Malaysia, 16150 Kubang Kerian, Kelantan, Malaysia; 2 Division of Occupational Medicine, Department of Community Dentistry, School of Dental Sciences, Health Campus, Universiti Sains Malaysia, 16150 Kubang Kerian, Kelantan, Malaysia; 3 School of Medicine and Health Sciences, Monash University, JKR 1235, Bukit Azah, 80100 Johor Bahru, Johor, Malaysia; 4 Institute of Medicine, Universiti Brunei Darussalam, Jalan Tungku Link, Gadong BE 1410, Brunei Darussalam

## Abstract

**Background:**

The relationships between working conditions [job demand, job control and social support]; stress, anxiety, and depression; and perceived quality of life factors [physical health, psychological wellbeing, social relationships and environmental conditions] were assessed using a sample of 698 male automotive assembly workers in Malaysia.

**Methods:**

The validated Malay version of the Job Content Questionnaire (JCQ), Depression Anxiety Stress Scales (DASS) and the World Health Organization Quality of Life-Brief (WHOQOL-BREF) were used. A structural equation modelling (SEM) analysis was applied to test the structural relationships of the model using AMOS version 6.0, with the maximum likelihood ratio as the method of estimation.

**Results:**

The results of the SEM supported the hypothesized structural model (*χ*^2 ^= 22.801, *df *= 19, *p *= 0.246). The final model shows that social support (JCQ) was directly related to all 4 factors of the WHOQOL-BREF and inversely related to depression and stress (DASS). Job demand (JCQ) was directly related to stress (DASS) and inversely related to the environmental conditions (WHOQOL-BREF). Job control (JCQ) was directly related to social relationships (WHOQOL-BREF). Stress (DASS) was directly related to anxiety and depression (DASS) and inversely related to physical health, environment conditions and social relationships (WHOQOL-BREF). Anxiety (DASS) was directly related to depression (DASS) and inversely related to physical health (WHOQOL-BREF). Depression (DASS) was inversely related to the psychological wellbeing (WHOQOL-BREF). Finally, stress, anxiety and depression (DASS) mediate the relationships between job demand and social support (JCQ) to the 4 factors of WHOQOL-BREF.

**Conclusion:**

These findings suggest that higher social support increases the self-reported quality of life of these workers. Higher job control increases the social relationships, whilst higher job demand increases the self-perceived stress and decreases the self-perceived quality of life related to environmental factors. The mediating role of depression, anxiety and stress on the relationship between working conditions and perceived quality of life in automotive workers should be taken into account in managing stress amongst these workers.

## Background

The influence of working conditions on health has been studied extensively over the last two decades [1-3]. The three most studied characteristics of the working conditions in relation to health are job demand, job control and social support at work [4]. The interaction of two factors – high job demand and low job control – in the job strain model [5] may predict adverse health effects on affected workers including fatigue, anxiety, depression, and physical illness [1]. An extension of this model – the iso-strain model (iso refers to social isolation) – posits that the most hazardous jobs occur when high job strain is combined with low levels of social support at work [6]. Most studies using this model have mainly focused on the relationship with cardiovascular diseases [2,7-9] and also musculoskeletal disorders [10], sickness absence [11], cigarette smoking [12] and general health outcomes such as self-reported health and quality of life (QOL) [13-15].

Stress, anxiety and depression have been recognized as important outcome measures in various work environments [16-18]. Plaisier et al. [19] suggested that poor working conditions may be an important precursor of stress and may, therefore, contribute to the development of depression or anxiety. There are abundant studies exploring the relationship between working conditions and stress, anxiety and depression [5,19-24]. Karasek et al. [5] have shown that workers with jobs simultaneously low in job control and high in job demand reported exhaustion, nervousness, anxiety, and insomnia or disturbed sleep. Sanne et al. [20] have also shown that job demand, job control and social support were independently associated with anxiety and depression. Meanwhile, Plaisier et al. [19] reported that job demand predicts the incidence of depressive and anxiety disorders in both men and women workers, but not for job control (decision latitude) and interaction between psychological demands and decision latitude.

Stress is often described as being associated with anxiety and depression at workplaces, and some studies have suggested that stress, anxiety and depression are also related to poor QOL [25-29]. Mendlowicz and Stein [30] suggested that anxiety disorders have a huge impact on the QOL. Recently, Chen et al. [26] reported that depressed subjects obviously had lower scores in every subscale of the health-related QOL than non-depressed subjects and suggested that depressed subjects were having poor QOL. Ravindran et al. [31] have also reported that major life stresses contribute toward depression, and depressive illness is often accompanied by marked reduction in QOL. However, the associations between anxiety and depression with QOL are still unclear due to very limited empirical data in the literature.

Although various studies have demonstrated the relationship between working conditions and depression and anxiety [19-21,23,32], awareness of the importance of QOL is also increasingly being recognized as an important relevant endpoint of outcome measure in diverse health populations including workers in stressful working conditions. Basically, QOL can be defined as an individual's perception of his/her position in life in the context of the culture and value systems in which he/she lives and in relation to his/her goals, expectations, standards and concerns. It is a broad ranging concept affected in a complex way by the person's physical health, psychological status, social relationships, and his/her environment [33]. Several studies have suggested the relationship between working conditions and worker's QOL [14,15,34,35]. For example, Nasermoaddeli et al. [34] have shown that low job demand is significantly associated with a higher perception of physical health, psychological status, and social relationship domains; whilst low job control (decision latitude) is related to the lower perception of the physical health domain of QOL. Meanwhile, Kudielka et al. [15] suggested that high job demand, low job control and lack of social support at work exert a significant impact on the self-reported health-related QOL.

Presently, no study has examined the relationship between working conditions and workers' QOL with stress, anxiety and depression as mediators. Since QOL is associated with stress, anxiety and depression [25-27,36], it is important to examine the role of stress, anxiety and depression variables as mediators. The aim of this study is, therefore, to investigate the relationship between job demand, job control, social support and stress, anxiety, depression and QOL, where, the effects of stress, anxiety and depression on QOL are also assessed simultaneously in this relationships using the structural equation modelling approach.

There were four hypotheses that have been developed and require further testing. Firstly, better working conditions (low job demand, high job control and high social support) are directly associated with higher perceptions of QOL domains (physical health, psychological status, social relationships and environment). Secondly, better working conditions (low job demand, high job control and high social support) are associated with lower stress, anxiety, and depression levels. Thirdly, lower stress, anxiety and depression levels are associated with higher perceptions of QOL domains (physical health, psychological status, social relationships and environment). Finally, a lower stress level is associated with a lower anxiety and depression level and plays an important role as a mediating factor in the relationship between better working conditions and the 4 domains of QOL (physical health, psychological status, social relationships and environment).

## Methods

### Study design

A cross-sectional study was conducted among male workers in two major representative automotive assembly plants located in Pahang and Selangor in Malaysia. This study is part of the Occupational Stress Intervention Study in Petroleum and Automobile Assembly Plants: Developing and Evaluating Stress Management Program at Workplaces (OSIS) for a period of three consecutive years beginning from July 2003.

### Research protocol

The study protocol was reviewed and approved by the Research and Ethics Committee, School of Medical Sciences, Universiti Sains Malaysia, Kelantan Health Campus. Permission to carry out the study was obtained from the Manager of Environmental Health and Safety Department, and Human Resource Department in each plant. The workers and employers were also given a written guarantee of confidentiality.

### Sample size

The estimation of sample size was performed using the single proportion formula with 95% confidence interval. Sample size calculation was based on the 50% prevalence of self-reported good or very good health status among Taiwanese male workers using the WHOQOL-BREF questionnaire [37]. We set the precision at 4% and the calculated sample size was 601. After considering a 15% non-response, the final calculated sample size was 691.

### Recruitment of study subjects

The automotive assembly industry was selected to represent the high income generating industries in Malaysia. The reference population consists of those workers in the paint shop and body shop in the automotive assembly plants in Malaysia. The source population includes workers in an automotive assembly plant in Selangor (plant A) and Pahang (plant B). The study population comprises 1100 workers for both plants – 800 in plant A (500 in the paint shop and 300 in the body shop) and 300 in plant B (200 in the paint shop and 100 in the body shop). The sampling method used for this study was universal. Permission to carry out the study was obtained from the Manager of Environmental Health and Safety Department and Human Resource Department in each plant. Inclusion and exclusion criteria were developed before recruiting the subjects. The inclusion criteria include male workers who are presently working in the paint shop and body shop and have worked for at least one year in the industry. Female workers and those serving less than one year were excluded. Workers were met at their worksites during working hours. The supervisors were asked to send their workers during the rest hour to the room set aside for data collection. Recruitment of workers was done using the list of workers provided by the supervisors; written informed consents were obtained from the workers participating in the study. Prior to completing the self-administered questionnaires (JCQ, DASS and WHOQOL-BREF), participating workers were given free medical check ups as an appreciation for their cooperation. Trained research officers checked the returned questionnaires onsite to ensure completeness. A total of 767 out of 1100 workers (response rate 69.72%) (521 in plant A and 246 in plant B) were recruited in the study. After excluding 39 female workers, the final study sample was 728 workers. However, due to 30 incomplete questionnaires discovered during data cleaning (missing data), only 698 subjects were included in the analysis.

### Working conditions

The validated Malay version of the Job Content Questionnaire [38] was used to measure working conditions that consist of three factors: job demand, job control (decision latitude) and social support at work based on the job strain model [1]. In this study, the job demand scale is the sum of 7 items – working fast, working hard, excessive work, not enough time, conflicting demands, intense concentration, hectic job and waiting on others; the Cronbach's alpha was 0.74. The job control (decision latitude) scale – refers to the person's ability to control his or her work activities [39] – is computed as the sum of 8 items: learning new things, requiring creativity, allows decision-making, high skill level, decision freedom, task variety, lots of say, and developing own abilities; the Cronbach's alpha was 0.61. Social support at work is the sum of 7 items – supervisor pays attention, helpful supervisor, friendly supervisor, a good organizer, co-workers competent, co-workers interest in me, and co-workers helpful; the Cronbach's alpha was 0.79. All items were scored on a Likert scale of 1 to 4 (1 = Strongly disagree, 2 = Disagree, 3 = Agree and 4 = Strongly agree; or 1 = Often, 2 = Sometimes, 3 = Rarely and 4 = Never). The scores for job control were reversed to allow for standardization of scores for job demand and social support.

### Stress, anxiety and depression

The validated Malay version of the Depression Anxiety Stress Scales (DASS) questionnaire was used to measure the negative self-perceived emotional states of stress, anxiety and depression [40]. Each of the three DASS scales contains 14 items, divided into subscales of 2–5 items with similar content. The DASS-Stress scale is sensitive to levels of chronic, non-specific arousal. It assesses difficulty relaxing, nervous arousal, and being easily upset/agitated, irritable/over reactive, and impatient; the Cronbach's alpha was 0.87. The DASS-Anxiety scale assesses autonomic arousal, skeletal muscle effects, situational anxiety, and subjective experience of anxious affect. For this scale, the Cronbach's alpha was 0.82. The DASS-Depression scale assesses dysphoria, hopelessness, devaluation of life, self-deprecation, lack of interest/involvement, anhedonia, and inertia; the Cronbach's alpha was 0.87. Subjects were asked to use a 4-point severity/frequency scale (0 = Did not apply to me at all, 1 = Applied to me to some degree, or some of the time, 2 = Applied to me a considerable degree, or a good part of the time, and 3 = Applied to me very much, or most of the time) to rate the extent to which they have experienced each state over the past week. Scores for DASS-Stress, DASS-Anxiety and DASS-Depression were calculated by summing the scores for the relevant items and converting these scores into percentile scores [41].

### Quality of life

The validated Malay version of the World Health Organization Quality of Life-Brief Version (WHOQOL-BREF) [42] is a 26-item version of the 100-item instrument of the World Health Organization Quality of Life (WHOQOL-100) that was developed to provide a short form QOL assessment concerned with the meaning of different aspects of life to the respondents, and how satisfactory or problematic are their experiences with these factors. It is a self-reported questionnaire containing four domains namely physical health (7 items), psychological status (6 items), social relationships (3 items) and environmental conditions (8 items). A previous validation study has shown that the Cronbach's alphas for the four domains of the WHOQOL-BREF were satisfactory (physical health = 0.80, psychological status = 0.64, social-relationships = 0.65 and environmental conditions = 0.73). All items were scored on a Likert scale of 1 to 5 (1 = Very poor, 2 = Poor, 3 = Neither poor nor good, 4 = Good, and 5 = Very good; 1 = Very dissatisfied, 2 = Dissatisfied, 3 = Neither satisfied nor dissatisfied, 4 = Satisfied, and 5 = Very satisfied; 1 = Not at all, 2 = A little, 3 = A moderate amount, 4 = Very much and 5 = An extreme amount; 1 = Not at all, 2 = A little, 3 = Moderately, 4 = Mostly, 5 = Completely; 1 = Not at all, 2 = A little, 3 = A moderate amount, 4 = Very much and 5 = Extremely; or 1 = Never, 2 = Seldom, 3 = Quite often, 4 = Very often and 5 = Always). The scores for some items were reversed to allow for comparison with other facets. The raw score of items within each domain is used to calculate the domain score by summing up the scores of the corresponding items in each domain. The domain score is then converted to a transformed score (ranging from 4 to 20) to enable comparison to be made between domains composed of unequal number of items. Domain scores were scaled in the positive direction, i.e. a higher score denotes a higher QOL.

### Statistical analysis

The data were analysed using the SPSS version 12.0.1 [43] and Analysis of Moment Structures (AMOS) version 6.0 [44,45]. A structural equation modeling (SEM), a type of multivariate analysis, was applied to confirm the theoretically built model that includes the domains of working conditions, stress, anxiety and depression, and consequently, QOL domains. In the first step, the model was designed and fitted with a well-defined research question. Secondly, the estimation and their significant levels for each parameter were obtained. Thirdly, model diagnostics including measures of model fitness and modification indices of AMOS, were obtained. If indicated, adding correlations between error terms and putting constraints on parameters were done to improve the model. The chi-square statistic provides a test of the null hypothesis that the theoretical model fits the data. Jöreskog and Sördom [46] suggested that the *p *value for this test of close fit should be more than 0.50. The criteria for model fit used were relative chi square statistic of less than or equal to 2.0 [45], Goodness-of-Fit Index (GFI) statistic of equal to or greater than 0.95 [46], Adjusted Goodness-of-Fit Index (AGFI) statistic of equal to or greater than 0.90 [45], Comparative Fit Index (CFI) of equal to or greater than 0.90 [47], and Root Mean Square Error of Approximation (RMSEA) of less than or equal to 0.8 [48]. A higher Parsimony Ratio (PRatio) suggests that the model is more parsimonious [49]. Finally, the estimates and significant levels of correlation and regression parameters from the fit model were presented. Total, direct and indirect effects of working conditions, stress, anxiety and depression to each QOL domain were calculated using the standardized regression weights of each pathway.

## Results

### Characteristics of observed variables

The characteristics of observed variables are shown in Table 1. The mean (SD) age of the workers was 27 (5.9) years. The mean (SD) educational level was 11.0 (1.4) years. The mean (SD) duration of work and salary were 6.1 (4.4) years and United States Dollar (USD) 386.1 (276.6), respectively. The mean (SD) job demand, job control and social support scores were 32.0 (3.8), 66.5 (7.8) and 27.5 (2.7), respectively. Meanwhile, the mean (SD) stress, anxiety and depression scores were 11.7 (6.8), 8.3 (5.5) and 8.3 (5.8), respectively. The mean (SD) physical health and psychological status scores were 14.7 (2.0) and 13.7 (1.7), respectively; and that for the environmental conditions and social relationships were 13.6 (1.9) and 14.8 (2.5), respectively.

**Table 1:** Demography, job content, self-perceived stress, anxiety and depression, and quality of life factors (n = 698)

**Variables**	**Mean**	**SD**
Demography		
1. Age (year)	27.3	5.9
2. Education (year)	11.0	1.4
3. Salary (USD)	386.1	276.6
4. Duration of work (year)	6.1	4.4
Job content		
5. Job demand	32.0	3.8
6. Job control	66.5	7.8
7. Social support	27.5	2.7
Self-perceived stress, anxiety and depression		
8. Stress	11.7	6.8
9. Anxiety	8.3	5.5
10. Depression	8.3	5.8
Self-perceived quality of life		
11. Physical health	14.7	2.0
12. Psychological status	13.7	1.7
13. Environmental conditions	13.6	1.9
14. Social relationships	14.8	2.5

### Correlation coefficients

Table 2 shows the Pearson correlation coefficient matrix of the observed variables. Job demand is inversely correlated with social support (*r *= -0.11, *p *< 0.01), physical health (*r *= -0.08, *p *< 0.05), and environmental conditions (*r *= -0.14, *p *< 0.01) and directly correlated with stress (*r *= 0.21, *p *< 0.01), anxiety (*r *= 0.18, *p *< 0.01) and depression (*r *= 0.19, *p *< 0.01). Job control is directly correlated with social support (*r *= 0.26, *p *< 0.01), physical health (*r *= 0.09, *p *< 0.05), psychological status (*r *= 0.09, *p *< 0.05), environmental conditions (*r *= 0.13, *p *< 0.01) and social relationships (*r *= 0.15, *p *< 0.01). Social support is directly correlated with physical health (*r *= 0.25, *p *< 0.01), psychological status (*r *= 0.20, *p *< 0.01), environmental conditions (*r *= 0.27, *p *< 0.01), and social relationships (*r *= 0.21, *p *< 0.01) and inversely correlated with stress (*r *= -0.21, *p *< 0.01), anxiety (*r *= -0.14, *p *< 0.01) and depression (*r *= -0.23, *p *< 0.01).

**Table 2:** Pearson correlation coefficient matrix of the measured variables

**Variables**	**1.**	**2.**	**3.**	**4.**	**5.**	**6.**	**7.**	**8.**	**9.**	**10.**	**11.**
1. Job demand	1										
2. Job control	-0.02	1									
3. Social support	-0.11**	0.26**	1								
4. Stress	0.21**	0.01	-0.21**	1							
5. Anxiety	0.18**	0.04	-0.14**	0.79**	1						
6. Depression	0.19**	-0.03	-0.23**	0.84**	0.74**	1					
7. Physical health	-0.08*	0.09*	0.25**	-0.41**	-0.40**	-0.39**	1				
8. Psychological status	-0.02	0.09*	0.20**	-0.22**	-0.19**	-0.27**	0.45**	1			
9. Environment	-0.14**	0.13**	0.27**	-0.34**	-0.27**	-0.33**	0.59**	0.54**	1		
10. Social relationships	-0.04	0.15**	0.21**	-0.29**	-0.23**	-0.29**	0.53**	0.45**	0.58**	1	
11. Age	0.04	0.10**	-0.15**	0.12**	0.13**	0.12**	-0.05	-0.08*	-0.01	0.12**	1

** Correlation is significant at the 0.01 level

* Correlation is significant at the 0.05 level

Stress is directly correlated with anxiety (*r *= 0.79, *p *< 0.01), and depression (*r *= 0.84, *p *< 0.01) and inversely correlated with physical health (*r *= -0.41, *p *< 0.01), psychological status (*r *= -0.22, *p *< 0.01), environmental conditions (*r *= 0.34, *p *< 0.01) and social relationships (*r *= 0.29, *p *< 0.01). Anxiety is directly correlated with depression and inversely correlated with physical health (*r *= -0.40, *p *< 0.01), psychological status (*r *= -0.19, *p *< 0.01), environmental conditions (*r *= -0.27, *p *< 0.01) and social relationships (*r *= -0.23, *p *< 0.01).

Depression is inversely correlated with physical health (*r *= -0.39, *p *< 0.01), psychological status (*r *= -0.27, *p *< 0.01), environmental conditions (*r *= -0.33, *p *< 0.01) and social relationships (*r *= -0.29, *p *< 0.01). Physical health is directly correlated with psychological status (*r *= 0.45, *p *< 0.01), environmental conditions (*r *= 0.59, *p *< 0.01) and social relationships (*r *= 0.53, *p *< 0.01). Psychological status is directly correlated with environmental conditions (*r *= 0.54, *p *< 0.01) and social relationships (*r *= 0.45, *p *< 0.01). Environment conditions is directly correlated with social relationships (*r *= 0.58, *p *< 0.01).

Age is directly correlated with job control (*r *= 0.10, *p *< 0.01), stress (*r *= 0.12, *p *< 0.01), anxiety (*r *= 0.13, *p *< 0.01), depression (*r *= 0.12, *p *< 0.01), social relationships (*r *= 0.12, *p *< 0.01) and inversely correlated with social support (*r *= -0.15, *p *< 0.01) and psychological status (*r *= -0.08, *p *< 0.05).

### Final model

**Figure 1:**
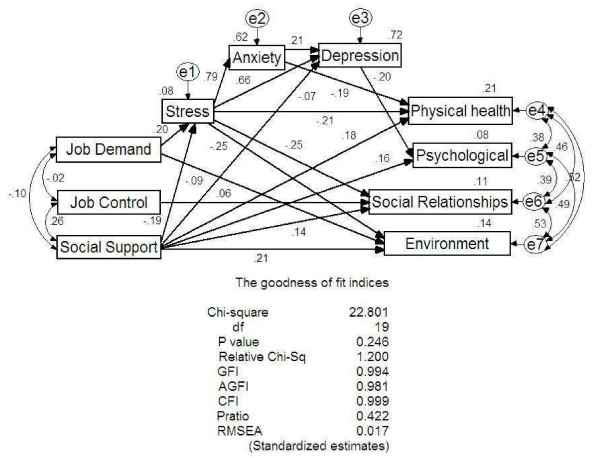
**Significant pathways of the final model and goodness-of-fit indices**. ^a ^e1–7 = error term 1–7. df = degree of freedom. GFI = Goodness-of-Fit Index. AGFI = Adjusted Goodness-of-Fit Index. CFI = Comparative Fit Index. Pratio = Parsimony Ratio. RMSEA = Root-Mean Square Error of Approximation.

Figure 1 shows significant pathways of the final model and their goodness-of-fit indices. The diagnostics of the model indicated that all error terms of QOL factors namely physical health, psychological status, environmental conditions and social relationships were inter-correlated. Similarly, the error terms of social support, job demand and job control were also inter-correlated. The measures of model fitness were as follows: chi square for Goodness-of-Fit test (*χ*^2 ^= 22.80, *df *= 19, *p *= 0.246), relative chi square (1.200), GFI (0.994), AGFI (0.981), CFI (0.999), RMSEA (0.017) and PRatio (0.442). All indices suggest that the presented final model reasonably fits the data.

### Significant relationships between observed variables

Table 3 shows the results of significant structural relationships between job demand, job control, social support and stress, anxiety, depression and QOL factors. Job demand is directly related to stress (*b *= 0.35; 95% CI: 0.22, 0.48; *p *< 0.001) and inversely related to environmental conditions (*b *= -0.09; 95% CI: -0.07, -0.02; *p *= 0.002). Job control is directly related to social relationships (*b *= 0.06; 95% CI: 0.22, 0.48; *p *< 0.001). Social support is inversely related to depression (*b *= -0.05; 95% CI: -0.22, -0.06; *p *= 0.001), stress (*b *= -0.19; 95% CI: -0.64, -0.29; *p *< 0.001), and directly related to physical health (*b *= 0.18; 95% CI: 0.08, 0.18; *p *< 0.001), psychological status (*b *= 0.16; 95% CI: 0.05, 0.15; *p *< 0.001), social relationships (*b *= 0.14; 95% CI: 0.06, 0.20; *p *< 0.001) and environment conditions (*b *= 0.21; 95% CI: 0.10, 0.19; *p *< 0.001).

**Table 3:** Relationship between independent and dependent variables

**Independent Variables**		**Dependent Variables**	***B*^a^**	***b*^b^**	**95% CI^c^**	***t *stat.**	***P value***
1. Job demand	--->	Stress	0.20	0.35	(0.22, 0.48)	5.36	<0.001
2. Job demand	--->	Environment	-0.09	-0.04	(-0.07, -0.02)	-3.16	0.002
3. Job control	--->	Social relationships	0.06	0.02	(0.00, 0.04)	2.10	0.036
4. Social support	--->	Depression	-0.05	-0.14	(-0.22, -0.06)	-3.23	0.001
5. Social support	--->	Stress	-0.19	-0.46	(-0.64, -0.29)	-5.11	<0.001
6. Social support	--->	Physical health	0.18	0.13	(0.08, 0.18)	5.33	<0.001
7. Social support	--->	Psychological status	0.16	0.10	(0.05, 0.15)	4.27	<0.001
8. Social support	--->	Social relationships	0.14	0.13	(0.06, 0.20)	3.85	<0.001
9. Social support	--->	Environment	0.21	0.14	(0.10, 0.19)	5.76	<0.001
10. Stress	--->	Anxiety	0.79	0.64	(0.60, 0.68)	33.67	<0.001
11. Stress	--->	Depression	0.66	0.56	(0.51, 0.62)	20.29	<0.001
12. Stress	--->	Physical health	-0.21	-0.06	(-0.09, -0.03)	-4.38	<0.001
13. Stress	--->	Social relationships	-0.25	-0.09	(-0.12, -0.06)	-7.02	<0.001
14. Stress	--->	Environment	-0.25	-0.07	(-0.09, -0.05)	-7.19	<0.001
Anxiety	--->	Depression	0.21	0.22	(0.15, 0.28)	6.39	<0.001
Anxiety	--->	Physical health	-0.19	-0.07	(-0.10, -0.04)	-4.23	<0.001
Depression	--->	Psychological status	-0.20	-0.06	(-0.08, -0.04)	-5.67	<0.001

^a ^*B *= Standardized regression Coefficient

^b ^*b *= Unstandardized regression Coefficient

^c ^95% CI of unstandardized regression Coefficient

Stress is directly related to anxiety (*b *= 0.79; 95% CI: 0.60, 0.68; *p *< 0.001), and depression (*b *= 0.66; 95% CI: 0.51, 0.62; *p *< 0.001); and inversely related to physical health (*b *= -0.21; 95% CI: -0.09, -0.03; *p *< 0.001), social relationships (*b *= -0.25; 95% CI: -0.12, -0.06; *p *< 0.001) and environmental conditions (*b *= -0.25; 95% CI: -0.09, -0.05; *p *< 0.001). Anxiety is directly related to depression (*b *= 0.21; 95% CI: 0.15, 0.28; *p *< 0.001) and inversely related to physical health (*b *= -0.19; 95% CI: -0.10, -0.04; *p *< 0.001). Depression is inversely related to psychological status (*b *= -0.20; 95% CI: -0.08, -0.04; *p *< 0.001).

### Mediating factors

Table 4 shows the total, direct and indirect effects of independent variables on the dependent variables. Stress, anxiety and depression are important mediators in the relationships between working conditions (job demand, job control, and social support) and QOL factors (physical health, psychological status, environmental conditions and social relationships). Job demand is indirectly related to physical health (mediated by stress and anxiety, *B *= -0.07); indirectly related to psychological status (mediated by stress, anxiety and depression, *B *= -0.03); both directly and indirectly related to environmental conditions (mediated by stress, total *B *= -0.14), and indirectly related to social relationships (mediated by stress, *B *= -0.05). Job control is directly related to social relationship (*B *= 0.06). Social support is both directly and indirectly related to physical health (mediated by stress and anxiety, total *B *= 0.25); both directly and indirectly related to psychological status (mediated by stress, anxiety and depression, total *B *= -0.17); both directly and indirectly related to environmental conditions (mediated by stress, total *B *= 0.25) and both directly and indirectly related to social relationships (mediated by stress, total *B *= 0.19).

**Table 4:** Total, direct and indirect effects of independent variables on dependent variables

	**Independent variables**						
**Dependent variables (QOL Factors)**	**Effect^a^**	**Job Demand**	**Job Control**	**Social Support**	**Stress**	**Anxiety**	**Depression**
1. Physical health	Total	-0.07	0.00	0.25	-0.36	-0.19	0.00
	Direct	0.00	0.00	0.18	-0.21	-0.19	0.00
	Indirect	-0.07	0.00	0.07	-0.15	0.00	0.00
2. Psychological status	Total	-0.03	0.00	0.20	-0.17	-0.04	-0.20
	Direct	0.00	0.00	0.16	0.00	0.00	-0.20
	Indirect	-0.03	0.00	0.04	-0.17	-0.04	0.00
3. Environmental conditions	Total	-0.14	0.00	0.25	-0.25	0.00	0.00
	Direct	-0.09	0.00	0.21	-0.25	0.00	0.00
	Indirect	-0.05	0.00	0.05	0.00	0.00	0.00

4. Social relationships	Total	-0.05	0.06	0.19	-0.25	0.00	0.00
	Direct	0.00	0.06	0.14	-0.25	0.00	0.00
	Indirect	-0.05	0.00	0.05	0.00	0.00	0.00

^a ^Standardized regression weight (*B*)

## Discussion

### Working conditions and quality of life

The relationship between working conditions and QOL have been reported in previous studies [14,15,34,35]. Important findings of this study reveal that social support is the most directly associated job factor with all domains of QOL. Hence, an increase in the social support at work predicts higher perceptions of the four dimensions of QOL. Previous studies have reported that poor social support is associated with lower perceptions of QOL. For instance, Cheng et al. [13] reported that poor social support in American women had a significant impact on poor functional status such as physical functioning, role limitations due to physical health problems, bodily pain, vitality, social functioning, and role limitations due to emotional problems, mental health and its decline over time. Other studies have also reported that low social support is associated with increased blood pressure [50], hyperlipidemia [51] and lower nutrient intake [52].

In addition, the mean age of the study population is relatively young. The matrix correlation analysis suggests that job control, stress, anxiety, depression and social relationships increase with increasing age; however, social support and psychological status decrease with increasing age. Ezoe et al. [53] reported that the grade of psychiatric symptoms measured by the Self-Rating Depression Scales, General Health Questionnaire, stress due to unsuitable jobs, and most of the DSM-III-R personality traits decreased with increasing age.

In the present study, job demand was inversely associated with the environmental conditions domain of QOL; whereas, job control was directly associated with the social relationships domain of QOL. These findings lend further support to our first hypothesis – favorable working conditions (low job demand, high job control and high social support) predict higher perceptions of QOL.

Various studies have also utilized the job demand, job control and social support model in conceptualizing the working conditions at workplaces in relation to the health-related QOL. Several studies categorize the job conditions into five: high strain, passive, low strain, active [14] and iso-strain [54]. Lerner et al. [14] have shown that high strain jobs (high job demand, low job control and low social support) are associated with poorer perceptions of all five components of the health-related QOL using the Medical Outcomes Study Short Form 12 Health Survey (SF12) instrument: physical functioning, role functioning related to physical health, vitality, social functioning, and mental health. Similarly, Amick et al.[54] reported that high strain jobs are associated with lower vitality, lower mental health, higher pain, and increased risks for both physical and emotional role limitations using the SF36 instrument. The present study also found that iso-strain is associated with an increased risk to the health of workers.

Cheng et al. [13] slightly adjusted the conceptualized model of the working conditions whereby job demand and job control were individually split into three categories: low, intermediate and high and social support into two categories: low and high. Based on these categories, women workers reporting high job demand and low job control (high strain job) were found to have the worst health status as assessed by a modified version of the SF-36, whereas those in jobs with high job control and low job demand (low strain job) had the best health status. Similarly, women workers reporting low job control, high job demand, and low social support were found to have a greater decline in their physical health subscales and less improvement in their mental health subscales.

Meanwhile, Kudielka et al. [15] conceptualized the working conditions as consisting of several factors: job demand, job control, social support and factors related to the effort-reward imbalance (ERI) model (effort and reward) as primary independent variables and are shown to be significantly associated with the mental summary score of the Short Form-12 Health Survey (SF12) including explaining 19% of the variance (*R*^2 ^= 19.0%). The variance for the working conditions decreases to 13% when accounting for demographics, socioeconomic status, body mass index, and medical condition. However, Nasermoaddeli et al., [34] who examined job demand and job control individually, found that job demand is independently and inversely related to physical health. On the other hand, job control is directly related to physical health, psychological health and social relationships domain of WHOQOL-BREF.

### Working conditions and stress, anxiety and depression

In the second hypothesis, we expect that poor working conditions (high job demand, low job control and poor social support) would be associated with higher self-perceptions of stress, anxiety, and depression. The present study shows that job demand is directly related to stress; however, job control is not related to stress, anxiety or depression. Wallgren and Hanse [55] surveyed 167 information technology (IT) consultants in Sweden and found that job demand is positively related to perceived stress. Their study also found that job control did not have a significant impact on stress. Whereas, Vanagas and Bihari-Axelsson [56] found that high job demand combined with low decision latitude have the greatest impact on stress among 300 Lithuanian General Practitioners.

The present study indicated that social support was inversely related to self-perceived stress and depression – high social support reduces self-perceived stress and depression. Thus, the second hypothesis was largely supported by the present study; only job control was not related to stress, anxiety or depression. Studies have also shown that social support has a protective effect on the development and prognosis of depression [19,57-59]. For example, Plaisier et al. [19] reported that high social support protected workers from depressive and anxiety disorders and buffered the unfavorable mental effect of working conditions. However, the beneficial effect of social support as reported might be limited to alexithymic individuals compared to the non-alexithymics. Thus, it would be worthwhile to attempt an intervention study that examines whether subjects who become less alexithymic after attending psychotherapy sessions that promote emotional awareness and non-verbal communications are able to utilize social support better than before, as suggested by Kojima et al. [60].

### Stress, anxiety and depression and quality of life

Two studies have reported that stress, anxiety and depression were related to poor QOL [25-29]. The third hypothesis maintains that lower stress, anxiety and depression levels are related to better QOL. The present study also found that self-perceived stress, anxiety and depression were inversely related to QOL factors, thus supporting our third hypothesis. Our data show that stress was inversely related to two QOL factors: environmental conditions and social relationships. Thus, lower self-perceived stress predicts higher perception of the QOL related to the environmental conditions and social relationships.

Our data show that self-perceived anxiety is inversely related to physical health, thereby suggesting that decreased anxiety predicts higher perception of the QOL related to physical health. In fact, there is evidence to suggest that anxiety disorders have a negative impact on QOL [30]. However, there is still a paucity of reports that could corroborate this finding. Therefore, it might be useful to examine the role of self-perceived anxiety in relation to QOL among workers.

Our data also show that self-perceived depression is inversely related to the psychological status domain of QOL: lower self-perceived depression predicts a higher perception of the QOL related to the psychological status domain. Chen et al. [26] demonstrated the negative effects of depression and physical illness on the scores of the QOL subscales of SF12 (mental and physical summary scores). Depressed police officers obviously had lower scores than non-depressed police officers in every subscale regardless of physical health. Those with physical illness showed lower scores on the subscale of physical illness, bodily pain, general health, and physical component summary.

### Mediating factors

SEM has supported our fourth hypothesis namely that stress, anxiety and depression act as mediators for the relationship between working condition factors and QOL factors. Stress mediates the relationship between job demand and physical health, job demand and social relationships, and job demand and environment. Similarly, self-perceived anxiety mediates the relationship between stress and depression, stress and physical health. Likewise, self-perceived depression demonstrated a mediating relationship in a number of studies. For example, Friedman et al.[61] predicted that depressed mood would mediate the relationship between social support and QOL. They found that the direct relationships between satisfaction and social support and both emotional and functional well-being were mediated by depression: less satisfaction with social support fostered depressed mood, which adversely impacted QOL.

We found that self-perceived anxiety was a mediator in the relationship between stress and depression. This result supports the model linking workload and depression as proposed by Greenglass et al. [62], who argue that distress at work, as operationalized by cynicism, emotional exhaustion, and anger is seen to lead towards depressed workload and depression. There are several methodological considerations that need to be taken into account when interpreting our results. Since our subjects were male automotive assembly plant workers in Malaysia, the generalizability of our results is limited. Since the study was cross-sectional, we cannot determine the directionality of the effects.

### Limitation of the study

Several limitations of this study should be noted. The cross-sectional design of our present study precludes any causal relationship between working conditions and stress, anxiety, depression and QOL. However, findings from other studies using longitudinal design, have suggested possible relationships between working conditions and stress, anxiety, and depression [63-68] and QOL [13,34,54]. To investigate issues of causality, future research could use prospective designs to replace the subjective responses related to the work environment in individuals with more objective measures using a job exposure matrix [69], standardized observations [70] and imputation techniques or observer-rating, together with self-report scales [71]. The present study also fully realized that questionnaires are not always reflecting the "true health". Thus, we suggest that further studies using physiological responses such as blood pressure and serum cortisol are needed to explore the relationship between working conditions and health. However, self-report is often the only feasible strategy to gather information concerning workers' working conditions [72]. Since various instruments were used to measure stress, anxiety and depression such as the Positive and Negative Affect Schedule (PANAS), Hospital Anxiety and Depression Scale (HADS), Beck Depression Inventory (BDI), Beck Anxiety Inventory (BAI) and QOL such as WHOQOL-BREF, Medical Outcomes Study Short-Form Health Survey 12 items (SF-12) and 36 items (SF-36) and other clinical diagnostic instruments in previous studies – our findings have limitations to be compared with many previous studies. Despite that, the mean age (27 years old) of our study sample was low. Kojima et al. [60] reported that the age range between 19 and 39 years are not in high demand nor in positions of high control in their workplaces, in comparison with those who are in their 40s or older. This age limitation might explain why our study found that job demand and job control were not significantly associated with all domains of QOL. Finally, the sample consisted of a specific group of workers, and one should therefore be cautious in generalizing the results beyond the study [73]. However, the usefulness of employing specific groups in working life to expand the understanding of the stress process has been emphasized [71].

## Conclusion

To our knowledge, our study is the first to outline the pattern of relationships between working conditions and stress, anxiety, depression and QOL by using SEM. The use of SEM permitted simultaneous evaluation of the effects of working condition on QOL; and stress, anxiety, depression on the QOL within the model. In addition, indirect effects could be assessed, which would have been impossible with standard regression methods. The results of the SEM (path analyses) supported the hypothesized model. Our study suggested that there are significant relationships between job demand, job control and social support with stress, anxiety and stress and QOL factors. Favorable job factors, especially high social support, play the most significant effect on all QOL factors (physical health, psychological status, environmental conditions and social relationships), stress and depression. Stress, anxiety and depression also have important roles in relation to QOL factors and should be taken into account in improving the QOL of workers.

## Competing interests

The author(s) declare that they have no competing interests.

## Authors' contributions

BNR, BAE and LN contributed equally to the design and conduct of the survey, analysis of the results, drafting and critical revision of the manuscript. BNR, BAE and LN read and approved the final version of the manuscript.

## Pre-publication history

The pre-publication history for this paper can be accessed here:


